# Clinical outcomes of initially asymptomatic patients with COVID-19: a Korean nationwide cohort study

**DOI:** 10.1080/07853890.2021.1884744

**Published:** 2021-02-13

**Authors:** Hayne Cho Park, Do Hyoung Kim, Ajin Cho, Juhee Kim, Kyu-sang Yun, Jinseog Kim, Young-Ki Lee

**Affiliations:** a Department of Internal Medicine, Kangnam Sacred Heart Hospital, Hallym University College of Medicine, Seoul, Korea; b Hallym University Kidney Research Institute, Seoul, Korea; c Department of Bigdata and Applied Statistics, Dongguk University, Gyeongju, Korea

**Keywords:** Asymptomatic infections, COVID-19, mortality, comorbidity

## Abstract

**Background:**

This study was performed to compare severe clinical outcome between initially asymptomatic and symptomatic infections and to identify risk factors associated with high patient mortality among initially asymptomatic patients.

**Methods:**

In this retrospective, nationwide cohort study, we included 5621 patients who had been discharged from isolation or died from COVID-19 by 30 April 2020. The mortality rate and admission rate to intensive care unit (ICU) were compared between initially asymptomatic and symptomatic patients. We established a prediction model for patient mortality through risk factor analysis among initially asymptomatic patients.

**Results:**

The prevalence of initially asymptomatic patients upon admission was 25.8%. The mortality rates were not different between groups (3.3% vs. 4.5%, *p* = .17). However, initially symptomatic patients were more likely to receive ICU care compared to initially asymptomatic patients (4.1% vs. 1.0%, *p* < .0001). The age-adjusted Charlson comorbidity index score (CCIS) was the most potent predictor for patient mortality in initially asymptomatic patients.

**Conclusions:**

The mortality risk was not determined by the initial presence of symptom among hospitalized COVID-19 patients. The CCIS was the most potent predictors for mortality. The clinicians should predict the risk of death by evaluating age and comorbidities but not the initial presence of symptom.

**Key messages**
- The mortality rate was not different between initially asymptomatic and symptomatic patients.
- Symptomatic patients were more likely to admitted to the intensive care unit.
- Age and comorbidities were the potent risk factors for mortality.

## Introduction

Since coronavirus disease 2019 (COVID-19) has been first reported in Wuhan, China in December 2019, it has been spread throughout the world with high infectivity. By 25 October 2020, over 42 million cases and 1.1 million deaths have been reported, and the average mortality rate has been reported around 2.73% [1,2]. The diagnosis of COVID-19 is based upon detection of nucleic acid of the severe acute respiratory syndrome coronavirus 2 (SARS-CoV-2) in patient samples by reverse transcriptase-polymerase chain reaction (RT-PCR) whether or not patients have clinical symptoms [3]. Therefore, COVID-19 infection includes both symptomatic and asymptomatic infections.

The incidence of asymptomatic infections has been varied because screening policies are different across countries [4]. The estimated proportion of asymptomatic infections ranges from 18% to 81% [5–7]. The difference in the incidence can be due to low awareness of asymptomatic infections in the early outbreak and limited detection capacity in some countries. In addition, the proportion of asymptomatic infection may be lower in hospitalized patients while it is higher in the re-tested positive cases [8].

Recently, the Centre for Disease Control and Prevention announced the revised guideline of testing for COVID-19. The guideline recommended COVID-19 testing for all symptomatic patients while limited testing for asymptomatic patients [9]. However, asymptomatic infections are known to have the same infectivity as symptomatic infections [10]. A recent paper demonstrated that viral load in asymptomatic patients were similar to that in symptomatic patients [11,12]. In addition, there is still the possibility for asymptomatic patients develop symptoms throughout disease course [5]. Therefore, early diagnosis of asymptomatic infections can be important to prevent further transmission of the viral disease.

Recent papers about asymptomatic patients with COVID-19 demonstrated that their clinical course is mild and most of them did not develop symptoms throughout isolation period [13,14]. However, there is no study about prognosis of initially asymptomatic carriers upon severe clinical outcome and mortality compared to symptomatic patients. Since Korean government not only performed confirmatory tests for those who are suspected to have the disease but also implemented screening tests for those without symptoms, asymptomatic and mild symptomatic cases were diagnosed in a large proportion. This study was performed among hospitalized patients during COVID-19 outbreak in Korea in order to demonstrate the difference in incidence of severe clinical outcome (mortality and admission to intensive care unit (ICU)) among initially asymptomatic patients compared to symptomatic patients and risk factors associated with severe outcome among initially asymptomatic patients.

## Materials and methods

### Study overview

This is a retrospective cohort study using the nationwide COVID-19 database provided by Korea Centres for Disease Control (KCDC). The database contains the clinical and epidemiologic data as well as clinical outcome of 5628 confirmed cases who had received treatment from 100 hospitals until 30 April 2020. Among them, 7 patients who were confirmed for COVID-19 after death were excluded, and a total of 5621 patients were included in the analysis. The mortality and ICU admission rates were compared between initially asymptomatic and symptomatic patients, and the risk factors associated with the outcomes were analysed.

### Definition

According to the definition provided by the KCDC [15], the confirmed case was defined as a patient who had been confirmed to be infected with COVID-19 by RT-PCR assay or virus isolation from nasal and/or pharyngeal swab specimens regardless of the clinical symptoms. Initially asymptomatic patients were defined as those without any symptoms at admission no matter what they developed symptoms during clinical courses. Anaemia was defined as a plasma haemoglobin (Hb) level of less than 120.0 g/L. Lymphocytopenia was defined as a lymphocyte count of less than 0.8 × 10^9^/L, and thrombocytopenia was defined as a platelet count of less than 150 × 10^9^/L.

### Data collection

Demographic data including age by decade, sex, survival status, ICU admission status, and duration of isolation were collected. The body mass index (BMI), systemic and diastolic blood pressure (BP), heart rate, and body temperature at initial visit were also measured and reported. The clinical symptoms associated with COVID-19 were reported among symptomatic patients including febrile sense, cough, sputum, sore throat, rhinorrhea, myalgia, shortness of breath, headache, altered consciousness/confusion, nausea/vomiting, and diarrhoea. Laboratory assessments consisted of plasma Hb, white blood cell, lymphocyte, and platelet count. The categories of comorbidities were assessed including diabetes mellitus, hypertension, heart failure, chronic heart disease, asthma, chronic obstructive pulmonary disease, chronic kidney disease, malignancy, chronic liver disease, connective tissue disease, and dementia. Age-adjusted Charlson comorbidity index score (CCIS) has been calculated from a weighted index consisted of age and the number and seriousness of comorbid diseases to predict the risk of mortality among COVID-19 patients [16,17]. Due to lack of available data, all kinds of chronic heart disease were considered to have congestive heart failure and all forms of chronic liver disease were considered to have mild liver disease. The presence of peripheral vascular disease, cerebrovascular disease, peptic ulcer disease, hemiplegia, end-stage renal disease, or AIDS could not be known from the current database. All kinds of malignancy were considered non-metastatic.

### Main outcomes

The primary outcome was defined as the patient mortality. The secondary outcome was the rate of ICU admission. Risk factors associated with outcomes were analysed and compared between initially asymptomatic and symptomatic patients. We established a prediction model for patient mortality through risk factor analysis among initially asymptomatic patients.

### Statistical analyses

The normally distributed numerical variables were expressed as the mean ± standard deviation, whereas variables with skewed distributions were expressed as the median and interquartile range. Statistical comparisons between continuous variables were performed with an independent Student *t*-test or one-way analysis of variance (ANOVA) for more than two groups. For the data without normal distribution, the Wilcoxon Signed Rank Test for two groups or Kruskal–Wallis Test for more than two groups were performed. Categorical measures are presented as percentages. The Chi square test and Fisher’s exact test were applied to categorical variables as appropriate.

The Kaplan–Meier method was used to compare death-free survival curves, and differences were assessed utilizing the log-rank test. We used univariate and multivariate Cox proportional hazard model to estimate risk factors associated with patient mortality. Age was excluded from the multivariate analysis because of its potential interaction with CCIS. We used univariate and multivariate logistic regression models to evaluate the risk factors for ICU admission. A nomogram to predict 14-day and 28-day mortality risk of the patient was built based on the variables found in multivariate Cox proportional hazard model. In the nomogram, CCIS was used as a continuous variable to check the impact per score. The maximum score of each variable was set as 100. The performance of the nomogram was measured based on the Harrell concordance index (C-index). The nomogram was validated in calibration plots with 1000 bootstrap samples in which the estimated survival probability was compared with the observed value. All statistical analysis was performed by using R version 4.0.2 (R Foundation for Statistical Computing; http://www.r-project.org/). *p* Value <.05 was considered statistically significant.

### Ethical statement

The present study protocol was reviewed and approved by the Institutional Review Board of the Kangnam Sacred Heart Hospital, Seoul, Korea (HKS 2020-06-025). The informed consent was waived due to retrospective nature of the study.

## Results

### Comparison of baseline characteristics between initially asymptomatic and symptomatic patients

In a total of 5621 patients, initially asymptomatic patients were 1449 (25.8%) and symptomatic patients were 4172 (74.2%). Baseline characteristics were compared between initially asymptomatic and symptomatic patients (Table 1). In the initially asymptomatic group, the proportions of patients under the age of 30 (30.0%) and over 70 years of age (17.5%) were greater than those in symptomatic group (22.9% and 14.5%, respectively) (Supplementary Figure 1). A total of 748 patients (51.6%) in initially asymptomatic group and 2556 patients (61.3%) in symptomatic group were female. The proportion of patients with low BMI (<18.5 kg/m^2^) was greater in initially asymptomatic group than that of symptomatic group. Initially asymptomatic patients had higher prevalence of dementia and lower prevalence of asthma than symptomatic group. In addition, the absolute lymphocyte and platelet counts were significantly lower in symptomatic patients compared to initially asymptomatic patients. In symptomatic patients, cough (56.1%) was the most common symptom followed by sputum (38.8%), febrile sense (31.2%), headache (23.2%), myalgia (22.2%), sore throat (21.1%), dyspnoea (15.9%), rhinorrhea (14.9%), diarrhoea (12.4%), nausea or vomiting (5.9%), fatigue (5.6%), and altered mentality (0.8%). The proportions of patients with no comorbidity (CCIS 0, 38.7% vs 31.2%) and high CCIS (≥5) (15.2% vs 11.9%) were greater in initially asymptomatic patients compared to symptomatic patients.

**Table 1.** Clinical characteristics between initially asymptomatic and symptomatic patients with COVID-19.

	Total (*n* = 5621) N (%)	Initially asymptomatic patients (*n* = 1449) N (%)	Initially symptomatic patients (*n* = 4172) N (%)	*p* Value
Age group, years				<.001
<10	66 (1.2)	32 (2.2)	34 (0.8)	
10–19	206 (3.7)	73 (5.0)	133 (3.2)	
20–29	1119 (19.9)	329 (22.7)	790 (18.9)	
30–39	564 (10.0)	147 (10.1)	417 (10.0)	
40–49	742 (13.2)	167 (11.5)	575 (13.8)	
50–59	1145 (20.4)	253 (17.5)	892 (21.4)	
60–69	914 (16.3)	194 (13.4)	720 (17.3)	
70–79	542 (9.6)	135 (9.3)	407 (9.8)	
≥80	323 (5.7)	119 (8.2)	204 (4.9)	
Male sex	2317 (41.2)	701 (48.4)	1616 (38.7)	<.001
Body mass index, kg/m^2^				.03
<18.5	260/4426 (5.9)	79/1092 (7.2)	181/3334 (5.4)	
≥18.5	4166/4426 (94.1)	1013/1092 (92.8)	3153/3334 (94.6)	
Systolic blood pressure, mmHg				.42
<120	1317/5486 (24.0)	347/1397 (24.8)	970/4089 (23.7)	
≥120	4169/5486 (76.0)	1050/1397 (75.2)	3119/4089 (76.3)	
Diastolic blood pressure, mmHg				.51
<80	2113/5486 (38.5)	549/1397 (39.3)	1564/4089 (38.2)	
≥80	3373/5486 (61.5)	848/1397 (60.7)	2525/4089 (61.8)	
Heart rate, beats/min	85.8 ± 15.1	84.4 ± 14.4	86.3 ± 15.3	<.001
Body temperature, °C	36.9 ± 0.6	36.7 ± 0.4	37.0 ± 0.6	<.001
Charlson comorbidity index score				<.001
0	1864 (33.2)	561 (38.7)	1303 (31.2)	
1–2	1692 (30.1)	378 (26.1)	1314 (31.5)	
3–4	1350 (24.0)	290 (20.0)	1060 (25.4)	
≥5	715 (12.7)	220 (15.2)	495 (11.9)	
Comorbidities				
Diabetes	689 (12.3)	178 (12.3)	511 (12.2)	>.99
Hypertension	1199 (21.3)	289 (19.9)	910 (21.8)	.15
Congestive heart failure	58 (1.0)	19 (1.3)	39 (0.9)	.28
Chronic heart disease	179 (3.2)	42 (2.9)	137 (3.3)	.53
Asthma	128 (2.3)	20 (1.4)	108 (2.6)	.01
Chronic obstructive pulmonary disease	40 (0.7)	10 (0.7)	30 (0.7)	>.99
Chronic kidney disease	55 (1.0)	18 (1.2)	37 (0.9)	.30
Malignancy	143 (2.5)	37 (2.6)	106 (2.5)	>.99
Chronic liver disease	82 (1.5)	22 (1.5)	60 (1.4)	.93
Connective tissue disease	38 (0.7)	6 (0.4)	32 (0.8)	.22
Dementia	224 (4.0)	112 (7.7)	112 (2.7)	<.001
Laboratory finding				
Lymphocyte count, ×10^9^/ L	1.69 ± 1.05	1.82 ± 0.92	1.65 ± 1.09	<.001
Haemoglobin, g/L	133 ± 18	133 ± 18	133 ± 17	.80
Platelet counts, ×10^9^/ L	236.9 ± 82.9	245.6 ± 85.1	234.0 ± 81.9	<.001

COVID-19: coronavirus disease 2019.

### Clinical outcomes according to the presence of initial symptoms

**Figure 1.**
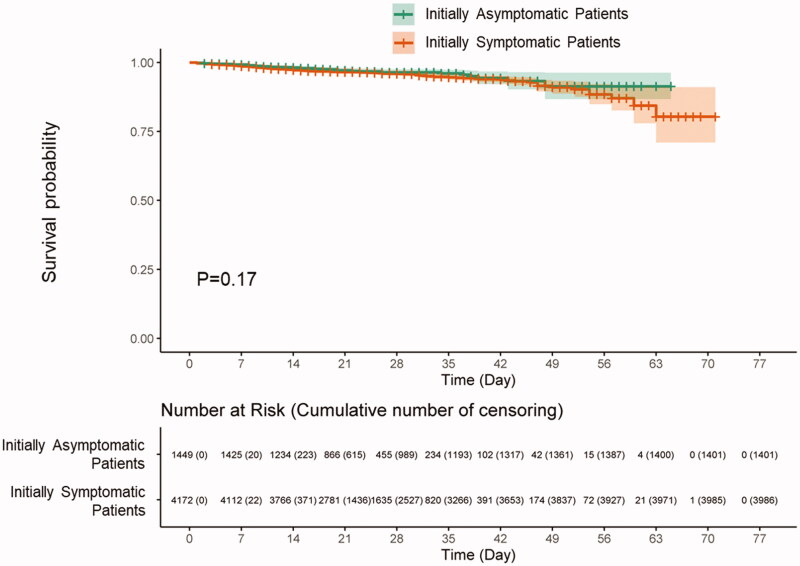
Kaplan–Meier survival plots for mortality according to the presence of initial symptoms. The figure displays the Kaplan–Meier survival plots of mortality between initially symptomatic and asymptomatic COVID-19 patients. There was no statistically significant difference between groups (*p* = .17).

Of the 234 patients (4.2%) who died during hospitalization, 48 (3.3%) were initially asymptomatic and 186 (4.5%) were symptomatic patients. The median duration from initial admission to death or release from the isolation treatment was 25.6 ± 11 .0 days. The mean follow-up duration was not statistically different between groups. In the Kaplan–Meier analysis, mortality rate was not statistically different between initially asymptomatic and symptomatic patients (*p* = .17; Figure 1). However, the patients with old age and high CCIS showed higher mortality rate in both symptomatic and initially asymptomatic groups (Supplementary Figure 2). Symptomatic patients with dementia, malignancy, connective tissue disease and diabetes had higher mortality compared to initially asymptomatic patients. On the other hands, initially asymptomatic patients with chronic obstructive pulmonary disease, chronic kidney disease and chronic heart disease showed higher mortality than symptomatic group. Table 2 showed the Cox proportional-hazards models for factors associated with in-hospital death. The male sex, low BMI (<18.5 kg/m^2^), high CCIS (≥3), anaemia, lymphocytopenia and thrombocytopenia were associated with high mortality. However, risk of death was not different according to the presence of symptom except dyspnoea.

**Table 2.** Predictors associated with mortality in the patients with COVID-19.

	Univariate		Multivariate	
	Hazard ratio (95% CI)	*p* Value	Hazard ratio (95% CI)	*p* Value
Age 50–69 years	14.00 (5.04–38.89)	<.001		
≥70 years	137.37 (51.00–370.03)	<.001		
Male sex	1.63 (1.26–2.11)	<.001	1.55 (1.05–2.29)	.03
Body mass inde*x* < 18.5 kg/m^2^	2.50 (1.48–4.22)	<.001	2.88 (1.66–4.99)	<.001
Systolic blood presssur*e* < 120 mmHg	1.18 (0.87–1.59)	.28		
Diastolic blood pressur*e* < 80 mmHg	1.36 (1.04–1.77)	.02	0.74 (0.50–1.09)	.13
Heart rat*e* ≥ 100 /min	1.74 (1.29–2.33)	<.001	1.07 (0.68–1.69)	.76
Body temperatur*e* ≥ 37.5 °C	2.13 (1.60–2.83)	<.001	1.18 (0.58–2.39)	.65
Charlson comorbidity index score ≥3	61.41 (27.29–138.15)	<.001	22.96 (7.20–73.24)	<.001
Any symptoms at admission	1.25 (0.91–1.72)	.17		
Febrile sense	2.02 (1.55–2.63)	<.001	1.31 (0.66–2.62)	.44
Fatigue	1.74 (1.06–2.85)	.028	1.15 (0.60–2.20)	.67
Dyspnoea	6.39 (4.94–8.26)	<.001	2.90 (1.96–4.29)	<.001
Altered mentality	19.77 (12.46–31.35)	<.001	2.03 (0.92–4.48)	.08
Haemoglobin <120 g/L	3.99 (3.07–5.19)	<.001	2.08 (1.40–3.11)	<.001
Lymphocyte count*s* < 0.8 × 10^9^/L	8.36 (6.41–10.89)	<.001	2.61 (1.75–3.89)	<.001
Platelet count*s* < 150 × 10^9^/L	3.95 (3.01–5.19)	<.001	2.03 (1.35–3.04)	<.001

CI: confidence interval; COVID-19: coronavirus disease 2019.

While there was no difference in mortality rate according to initial presence of symptoms, symptomatic patients were more likely to be admitted to ICU compared to initially asymptomatic patients. A total of 172 (4.1%) symptomatic patients were admitted to ICU while only 15 (1.0%) initially asymptomatic patients were during hospitalization. In multivariate analysis, male sex, high CCIS (≥3), anaemia, and lymphocytopenia were related to ICU admission (Supplementary Table 1). In addition, dyspnoea (hazard ratio, 4.65; 95% CI, 3.13–6.90) and altered mentality (hazard ratio, 6.06; 95% CI 1.56–23.50) were risk factors of ICU admission.

### Risk factors for severe clinical outcomes in initially asymptomatic patients

We assessed the risk factors for mortality in initially asymptomatic patients. In multivariate analysis, high CCIS (≥5) was an independent risk factor for mortality (hazard ratio, 61.91; 95% CI 8.29–462.62) together with lymphocytopenia (hazard ratio, 2.46; 95% CI 1.18–5.11) and thrombocytopenia (hazard ratio, 2.60; 95% CI 1.31–5.19) (Table 3).

**Table 3.** Cox proportional analysis of predictors associated with mortality in initially asymptomatic patients with COVID-19.

	Univariate		Multivariate	
	Hazard ratio (95% CI)	*p* Value	Hazard ratio (95% CI)	*p* Value
Ag*e* ≥ 70 years	30.98 (13.16–72.94)	<.001		
Male sex	0.69 (0.38–1.22)	.20		
Body mass inde*x* < 18.5 kg/m^2^	2.56 (0.56–11.61)	.22		
Systolic blood pressur*e* < 120 mmHg	1.23 (0.63–2.39)	.54		
Diastolic blood pressur*e* < 80 mmHg	1.12 (0.62–2.03)	.71		
Heart rat*e* ≥ 100/min	1.13 (0.51–2.54)	.76		
CCIS 3–4	22.20 (2.73–180.44)	.004	6.58 (0.73–59.35)	.09
≥5	158.80 (21.82–1155.85)	<.001	61.91 (8.29–462.62)	<.001
Diabetes	4.54 (2.54–8.10)	<.001		
Hypertension	4.99 (2.82–8.83)	<.001		
Congestive heart failure	8.79 (3.47–22.27)	<.001		
Chronic heart disease	6.64 (3.10–14.19)	<.001		
Asthma	2.81 (0.68–11.58)	.15		
Chronic pulmonary obstructive disease	19.71 (7.74–50.22)	<.001		
Chronic kidney disease	12.83 (5.43–30.30)	<.001		
Malignancy	1.82 (0.44–7.51)	.41		
Chronic liver disease	1.64 (0.23–11.91)	.63		
Dementia	11.15 (6.32–19.69)	<.001		
Haemoglobin <120 g/L	5.42 (2.95–9.95)	<.001	1.55 (0.81–2.94)	.19
Lymphocyte count*s* < 0.8 × 10^9^/L	5.53 (2.85–10.73)	<.001	2.46 (1.18–5.11)	.02
Platelet count*s* < 150 × 10^9^/L	4.70 (2.53–8.76)	<.001	2.60 (1.31–5.19)	.007

CCIS: age-adjusted Charlson comorbidity index score; CI: confidence interval; COVID-19: coronavirus disease 2019.

**Figure 2.**
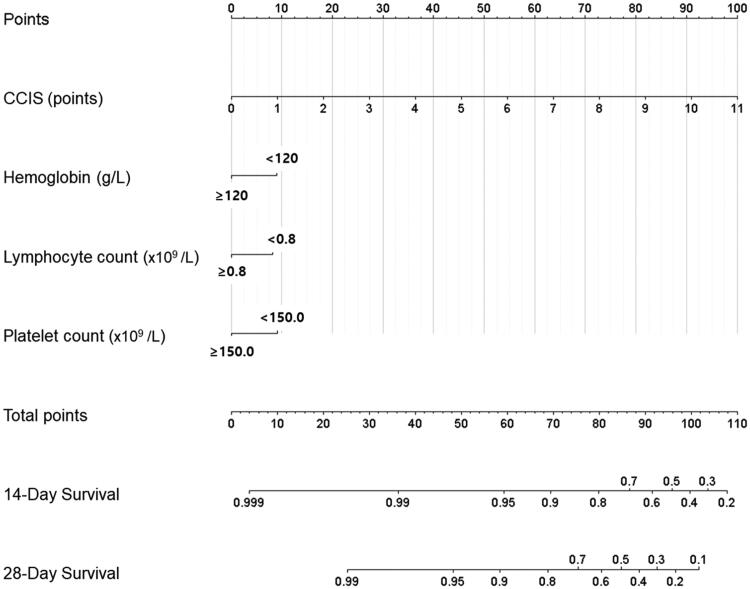
Prognostic nomogram for prediction of the overall survival probability of initially asymptomatic patients with COVID-19. The nomogram demonstrates Charlson comorbidity index score is the potent predictor for 14-day and 28-day survival of the patients.

The predictive nomogram was constructed based on the multivariate Cox analysis for mortality. The 14- and 28-day overall survival probability was calculated from the summed points of CCIS, anaemia, lymphocytopenia, and thrombocytopenia (Figure 2). The nomogram showed that CCIS was the most important factor contributing to the prognosis followed by the thrombocytopenia, lymphocytopenia, and anaemia. The calibration plots on bootstrap resampling validation are shown in the Supplementary Figure 3. The C-index value for prediction of overall survival was 0.892, and R^2^ value was 0.992 in 14-day and 0.997 in 28-day prediction model.

## Discussion

This study examined the mortality and ICU admission rates among initially asymptomatic and symptomatic patients during hospitalization due to COVID-19 infection in Korea. The mortality rate in initially asymptomatic patients was comparable to that in symptomatic patients (3.3% vs 4.5%, *p* = .17). However, admission rate to ICU was greater in symptomatic patients compared to that in initially asymptomatic patients (4.1% vs 1.0%, *p* < .001). The CCIS was the most potent predictor for mortality in initially asymptomatic patients. 

Previous studies were performed to explain clinical course and outcomes of COVID-19 among hospitalized patients [18–20]. In most of the countries, screening tests for COVID-19 are performed against those who developed symptoms. Recent guideline by the Centres for Disease Control and Prevention also did not recommend the COVID-19 test for asymptomatic patients who are in close contact with the confirmed case. However, there has been no evidence about whether symptomatic and asymptomatic patients should be dealt differently from screening, diagnosis, and treatment. There has been no study comparing clinical outcome between initially asymptomatic and symptomatic patients.

To our knowledge, this is the first study to evaluate incidence of mortality and its risk factors in initially asymptomatic patients upon admission. Interestingly, the presence of symptom at diagnosis was not as important as age or comorbidities in prediction of patient mortality. However, symptomatic patients at admission were more likely to receive ICU care. Previous study inferred that patients may spread virus 1–3 days before symptom development, and the duration of infectious period may be 6.5–9.6 days in asymptomatic patients [21]. Another recent article demonstrated that the cycle threshold values in asymptomatic patients were similar to those in symptomatic patients, which suggest that asymptomatic patients have similar infectious capacity compared to symptomatic patients [22]. On the other hands, the clinical severity of COVID-19 may be related to viral load. Recent brief article by Liu *et al.* demonstrated that the patients with severe COVID-19 tend to have a high viral load and a long viral shedding period compared to the patients with mild COVID-19 [23]. Another article by German researchers found that the duration of viral shedding was not related to viral replication and isolation from tissue [24]. Therefore, a complex dynamic between the virus and immune reaction of the host may underlie the severity of the disease and clinical course of COVID-19. In that venue, it is not surprising to find that symptomatic patients were more likely to show severe clinical course and receive ICU care during hospitalization.

However, the mortality was not determined by the initial presence of symptoms. Compared to symptomatic patients, initially asymptomatic patients in our cohort demonstrated greater proportion of elderly population, low BMI, male prevalence, greater proportion of high CCIS (≥5) and higher prevalence of dementia. On the other hands, the degree of lymphocytopenia and thrombocytopenia, which are associated with clinical severity of the disease, were milder in initially asymptomatic patients. Previous papers well documented the effect of comorbidities or CCIS upon case-fatality rate. In Italy where one of the highest case-fatality rates were reported, the non-survivor demonstrated advanced age and higher CCIS compared to survivors [18]. Chinese researchers suggested predictive nomogram for fatal outcome including age and pre-existing comorbid conditions [19]. Therefore, the host factors including age and comorbidities are more important in determining the case-fatality rate than the virus factors or the presence and severity of symptoms.

There are some limitations to our study. First, there can be an inherent risk for selection bias as the indication for testing may vary between initially asymptomatic cases and symptomatic cases. Although some adjustment for other factors was performed, not all information regarding potential confounders were available in our study. In addition, we excluded those who admitted to the community treatment centres and only included the hospitalized patients. Therefore, we may not generalize our results. However, in the initial period of COVID-19 pandemic in Korea, most of the patients were initially admitted to the hospital irrespective of clinical severity or presence of symptoms. Therefore, our results may represent the general aspects of Korean COVID-19 patients. Second, initially asymptomatic patients may include both presymptomatic patients and asymptomatic patients [5]. Therefore, some of them would have developed symptoms at some time after admission. The previous study by Korean researchers demonstrated that about one-third of initially asymptomatic patients developed symptoms during clinical course [13]. Therefore, we cannot conclude from our study that the prognosis of asymptomatic patients during entire clinical course is similar to that of those who developed symptoms afterwards. Recent review article by Berlin *et al.* demonstrated that severe illness usually begins approximately 1 week after the onset of symptoms [25]. Therefore, similar mortality rate of initially asymptomatic patients with symptomatic patients may result from subsequent development of symptoms and severe illness among initially asymptomatic population. Lastly, the patients with dementia and chronic obstructive pulmonary disease or asthma may have under-reported their symptoms, which may result in overestimation of asymptomatic patients.

Overall, we found that mortality risk was not determined by the initial presence of symptoms in the patients with COVID-19. Our study suggests that initially asymptomatic patients should not be considered “less severe” than symptomatic patients in treating COVID-19. Regardless of the presence of symptoms, we should predict the clinical risks of the patients based upon age and comorbidities and treat them accordingly.

## Supplementary Material

Supplemental Material

## Data Availability

Raw data were generated at Korean Centres for Disease Control and Prevention. Derived data supporting the findings of this study are available from the corresponding author (Y.K.L.) on request.
