# Supplementary material for: Clinical outcomes of initially asymptomatic patients with COVID-19: a Korean nationwide cohort study
Source: Ann Med. 2021 Feb 13;53(1):357–64. doi: 10.1080/07853890.2021.1884744 (PMC7889197; doi:10.1080/07853890.2021.1884744)
Supplement: Supplemental Material [file IANN_A_1884744_SM1467.zip › suppl_data/SupplementaryFigure1.docx]

**Supplementary Figure 1. Age distribution of the patients with COVID-19 according to the initial presence of symptoms.** In the initially asymptomatic group, the proportions of patients under the age of 30 (30.0%) and over 70 years of age (17.5%) were greater than those in symptomatic group (22.9% and 14.5%, respectively). Abbreviation: COVID-19, coronavirus disease 2019.

**
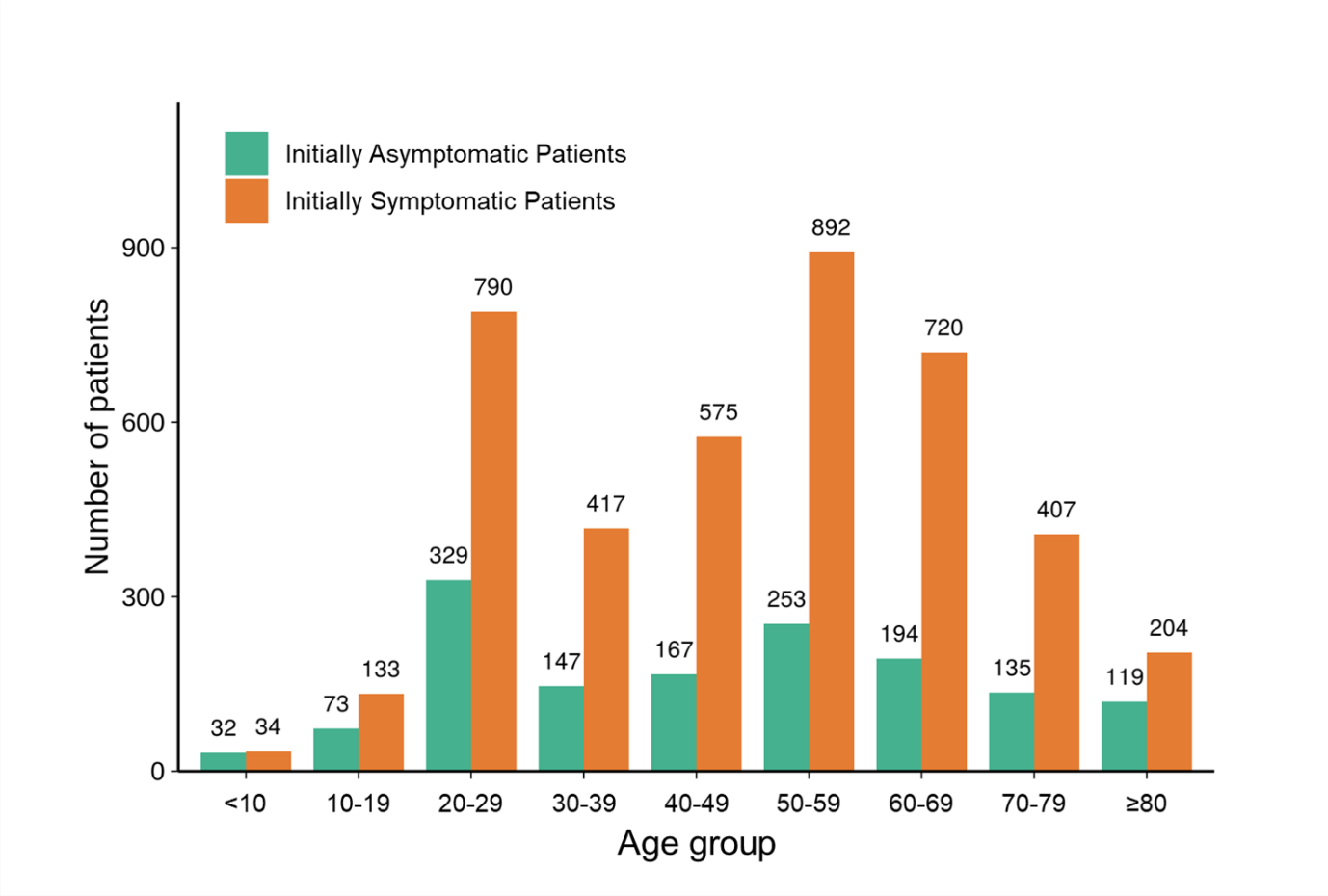
**
