# Supplementary material for: Clinical outcomes of initially asymptomatic patients with COVID-19: a Korean nationwide cohort study
Source: Ann Med. 2021 Feb 13;53(1):357–64. doi: 10.1080/07853890.2021.1884744 (PMC7889197; doi:10.1080/07853890.2021.1884744)
Supplement: Supplemental Material [file IANN_A_1884744_SM1467.zip › suppl_data/SupplementaryFigure2.docx]

**Supplementary Figure 2. Percentage of death according to (A) age, (B) CCIS, and (C) comorbidities.** The patients with old age and high CCIS showed higher mortality rate in both initially symptomatic and asymptomatic groups. Symptomatic patients with dementia, malignancy, connective tissue disease and diabetes had higher mortality compared to initially asymptomatic patients. On the other hands, initially asymptomatic patients with chronic obstructive pulmonary disease, chronic kidney disease and chronic heart disease showed higher mortality than symptomatic group. Abbreviation: CCIS, Charlson comorbidity index score; CHF, Congestive heart failure; CKD, Chronic kidney disease; CLD, Chronic liver disease; COPD, Chronic obstructive pulmonary disease; CTD, Connective tissue disease; HTN, hypertension.

**
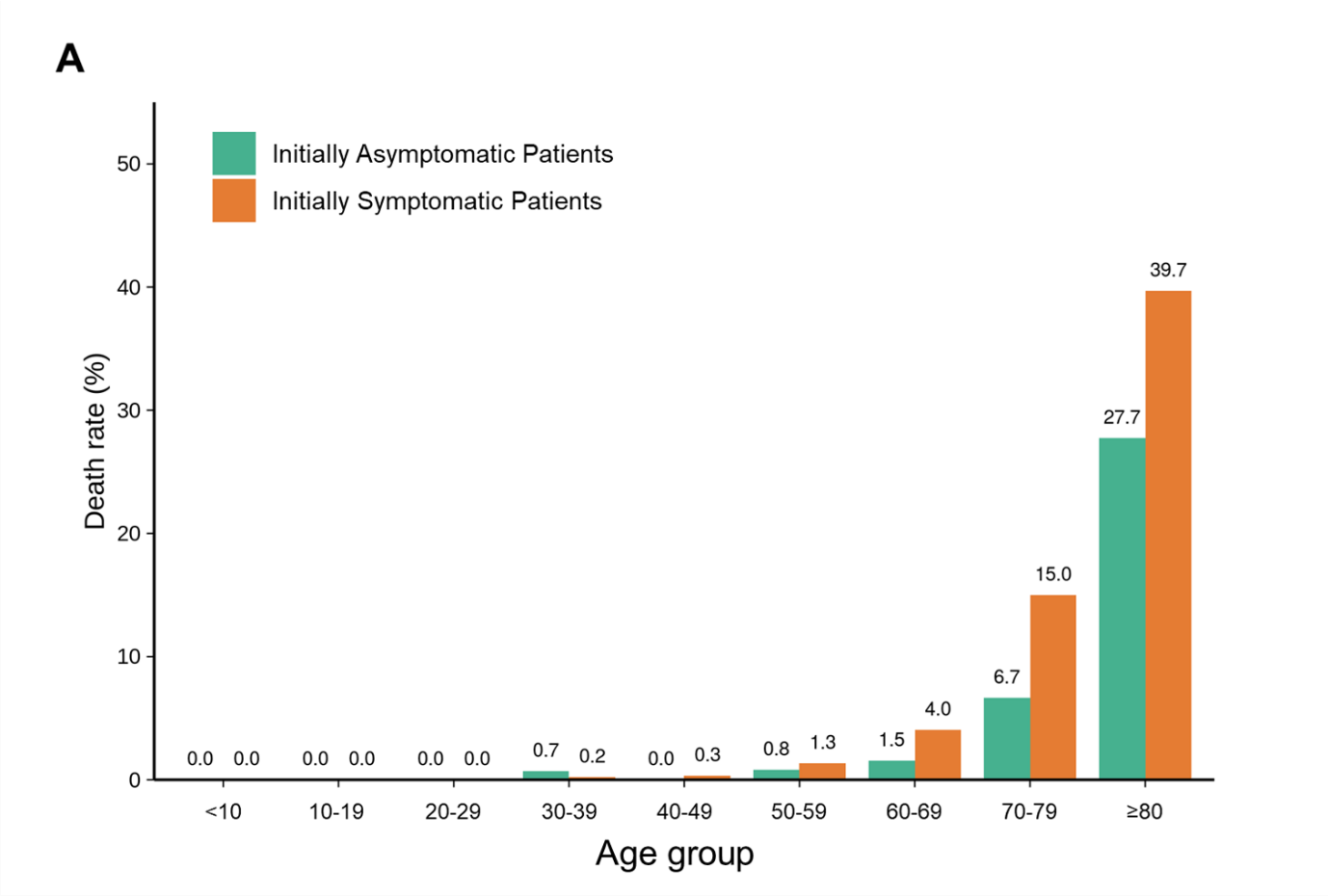
**

**
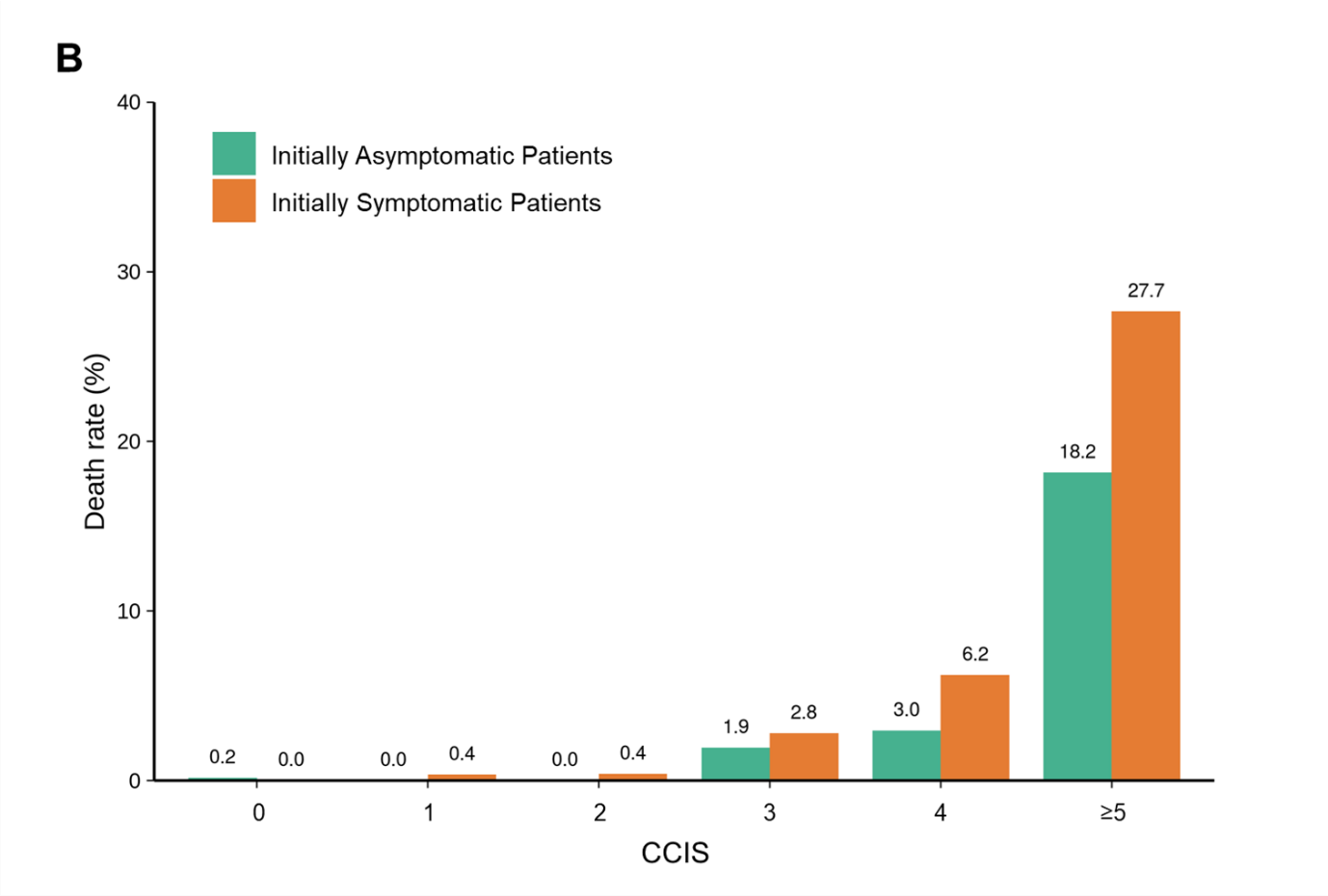
**

**
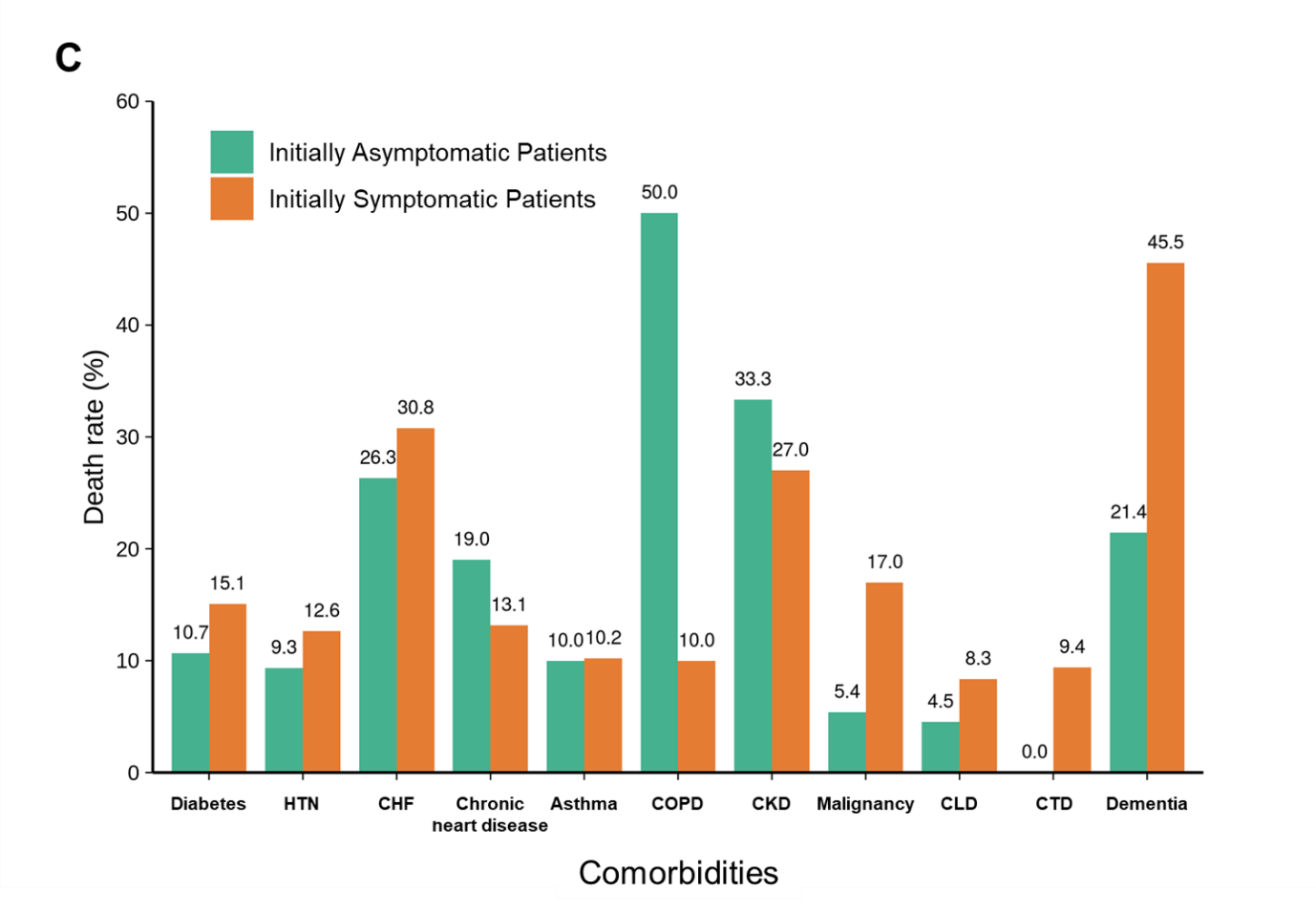
**
