# Supplementary material for: Clinical outcomes of initially asymptomatic patients with COVID-19: a Korean nationwide cohort study
Source: Ann Med. 2021 Feb 13;53(1):357–64. doi: 10.1080/07853890.2021.1884744 (PMC7889197; doi:10.1080/07853890.2021.1884744)
Supplement: Supplemental Material [file IANN_A_1884744_SM1467.zip › suppl_data/SupplementaryFigure3.docx]

**Supplementary Figure 3. Calibration curves of the nomogram predicting (A) 14-day and (B) 28-day overall survival in asymptomatic patients with COVID-19.** Abbreviation: COVID-19, coronavirus disease 2019.

**
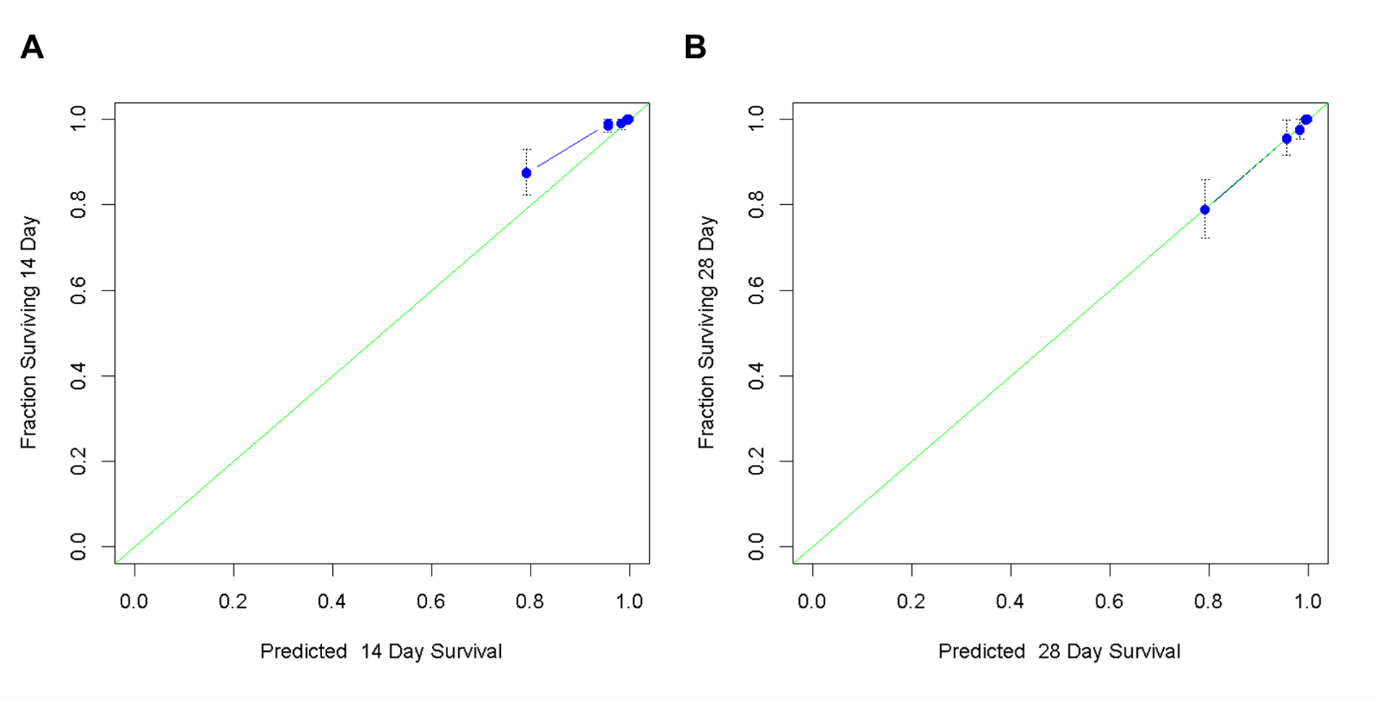
**
