# Supplementary material for: Clinical outcomes of initially asymptomatic patients with COVID-19: a Korean nationwide cohort study
Source: Ann Med. 2021 Feb 13;53(1):357–64. doi: 10.1080/07853890.2021.1884744 (PMC7889197; doi:10.1080/07853890.2021.1884744)
Supplement: Supplemental Material [file IANN_A_1884744_SM1467.zip › suppl_data/SupplementaryTable1.docx]

**Supplementary Table 1. Univariate and multivariate logistic regression analysis of predictors for admission to intensive care unit in the patients with COVID-19**

|  | **Univariate** | | **Multivariate** | |
| --- | --- | --- | --- | --- |
|  | **Odd ratio (95% CI)** | ***P* value** | **Odd ratio (95% CI)** | ***P* value** |
| Age 50-69 years | 4.69 (2.88 - 7.65) | <0.001 |  |  |
| ≥70 years | 15.27 (9.45 - 24.68) | <0.001 |  |  |
| Male | 2.34 (1.74 - 3.16) | <0.001 | 2.45 (1.63 - 3.67) | <0.001 |
| Body mass index <18.5 kg/m^2^ | 0.92 (0.45 - 1.90) | 0.82 | 0.85 (0.37 - 1.98) | 0.71 |
| Systolic blood pressure <120 mmHg | 0.76 (0.53 - 1.10) | 0.15 |  |  |
| Diastolic blood pressure <80 mmHg | 1.31 (0.98 - 1.76) | 0.07 | 1.25 (0.84 - 1.86) | 0.26 |
| Heart rate ≥100/min | 1.89 (1.36 - 2.63) | <0.001 | 1.40 (0.88 - 2.22) | 0.16 |
| Body temperature ≥37.5℃ | 3.63 (2.68 - 4.92) | <0.001 | 1.3 (0.68 - 2.74) | 0.38 |
| CCIS ≥3 | 8.28 (5.68 - 12.05) | <0.001 | 3.63 (2.24 - 5.88) | <0.001 |
| Any symptoms at admission | 4.12 (2.42 - 7.00) | <0.001 |  |  |
| Febrile sense | 3.52 (2.62 - 4.72) | <0.001 | 1.92 (0.99 - 3.74) | 0.06 |
| Fatigue | 2.41 (1.44 - 4.05) | <0.001 | 1.17 (0.59 - 2.32) | 0.66 |
| Dyspnea | 9.00 (6.67 - 12.14) | <0.001 | 4.65 (3.13 - 6.90) | <0.001 |
| Altered mentality | 16.01 (7.60 - 33.73) | <0.001 | 6.06 (1.56 - 23.50) | 0.009 |
| Hemoglobin <120 g/L | 2.36 (1.72 - 3.26) | <0.001 | 2.14 (1.38 - 3.31) | <0.001 |
| Lymphocyte counts <0.8*10^9^/L | 6.68 (4.83 - 9.25) | <0.001 | 1.70 (1.08 - 2.65) | 0.02 |
| Platelet counts <150*10^9^/L | 3.05 (2.16 - 4.31) | <0.001 | 1.34 (0.84 - 2.14) | 0.22 |

Abbreviation: CCIS, age-adjusted Charlson comorbidity index score; CI, confidence interval; COVID-19, coronavirus disease 2019.
